# An integrated clinical and imaging model for predicting post-traumatic nonunion

**DOI:** 10.3389/fmed.2026.1784029

**Published:** 2026-04-13

**Authors:** Bin Wang, Kaipan Qu

**Affiliations:** 1 Navy 971st Hospital of PLA, Qingdao, China; 2 Department of Trauma and Hand Foot Surgery, Shandong Provincial Third Hospital, Jinan, China

**Keywords:** image, machine learning, post-traumatic nonunion, prediction model, SHAP

## Abstract

**Objective:**

This model aims to support early risk stratification and clinical decision-making.

**Methods:**

A retrospective cohort of 343 patients with unilateral closed long bone fractures treated by internal fixation was analyzed. Clinico-radiological variables—including Injury Severity Index, maximum fracture gap width, volume of cystic changes, callus volume growth rate, and Radiographic Union Scale for Tibial fractures (RUST) score—were systematically collected. Predictors were selected using Least Absolute Shrinkage and Selection Operator (LASSO) regression. Multivariable logistic regression and multiple machine learning algorithms (e.g., random forest, gradient boosting machine) were applied. The dataset was split 7:3 into training and validation sets. All feature selection and model hyperparameter tuning were restricted to the training set, information leakage was avoided via 10-fold cross-validation, and a single evaluation was finally conducted on the independent validation set. Model performance was assessed using the area under the receiver operating characteristic curve (AUC), calibration curves, and decision curve analysis.

**Results:**

Training and validation cohorts exhibited comparable baseline characteristics. Five independent predictors were identified: Injury Severity Index, maximum fracture gap width, and cystic change volume were risk factors, whereas callus growth rate and RUST score were protective. The gradient boosting machine model achieved the highest predictive accuracy, with an AUC of 0.866 (95% Confidence Interval (CI): 0.783–0.948) in training and 0.858 (95% CI: 0.716–1.000) in validation. Calibration was satisfactory, and decision curve analysis demonstrated a superior net benefit across threshold probabilities. A nomogram was constructed, and SHapley Additive exPlanations (SHAP) analysis improved interpretability.

**Conclusion:**

A machine learning model integrating clinical and imaging predictors was successfully developed and validated for predicting post-traumatic nonunion. It exhibits strong discriminative ability and may assist in personalized treatment planning.

## Introduction

Fractures are common clinical injuries. The majority of fractures heal successfully with standard treatment. However, a subset of cases progresses to nonunion, leading to prolonged pain, functional impairment, and multiple surgical interventions, thereby imposing a significant burden on patients and society (1, 2). The occurrence of post-traumatic nonunion results from the interplay of multiple factors. Traditional perspectives have primarily focused on clinical risk factors such as smoking, diabetes, and infection, as well as treatment-related factors including fracture type and fixation method (3, 4). However, these factors largely assess initial conditions and systemic status, failing to dynamically and precisely capture local biological changes during the healing process.

Recent advances in imaging technology have provided new perspectives for evaluating the biological progression of fracture healing through functional imaging and quantitative analysis. For instance, CT-based three-dimensional callus volume measurement, microcirculatory parameters (e.g., Ktrans value) from dynamic contrast-enhanced magnetic resonance imaging (DCE-MRI), and bone metabolic activity assessment via Single-Photon Emission Computed Tomography / Computed Tomography (SPECT/CT) can objectively quantify the healing potential and failure risk at the fracture site across structural, vascular, and metabolic dimensions (5, 6). Currently, clinical decision-making still heavily relies on physician experience, lacking an objective, quantitative tool that integrates multi-dimensional information for early, individualized risk stratification.

Machine learning, a powerful data analysis method capable of handling complex variables with high-dimensional, non-linear relationships, demonstrates considerable potential in building medical prediction models (7). It can identify key patterns from vast amounts of clinical and imaging data that are difficult for the human brain to discern, enabling more accurate predictions. Therefore, developing a machine learning prediction model that integrates key clinical risk factors with multi-modal quantitative imaging features is crucial for the early identification and intervention of post-traumatic nonunion.

This study aims to systematically collect clinical data and serial imaging data from patients with traumatic fractures, extract key clinical risk indicators and quantitative imaging features, and utilize machine learning algorithms to construct and validate a comprehensive prediction model for post-traumatic nonunion. The goal is to provide clinicians with an objective decision-support tool and contribute to the precise management of fracture treatment.

## Materials and methods

### Study population

This retrospective cohort study aimed to develop and validate a prediction model for post-traumatic nonunion. We prospectively collected clinical and imaging data from adult patients with unilateral, closed long bone fractures (including tibia, femur, and humerus) who were admitted to our hospital’s Department of Orthopedics and underwent surgical internal fixation between January 2022 and June 2024. The study protocol was approved by our hospital’s Ethics Committee.

Sample size calculation was based on an expected incidence of the primary outcome (nonunion) of 15%–20% [according to our center’s preliminary data and international literature (8, 9)]. The calculation was performed using methods appropriate for the core statistical analyses (multivariable logistic regression and machine learning algorithms). Verification was conducted using G*Power 3.1.9.7 software and the “pwr” package in R 4.3.1: significance level *α* = 0.05 (two-tailed), test power 1–β = 80%, and approximately 15–20 candidate predictor variables. The minimum required number of events (nonunion cases) was estimated as 10–15 times the number of predictor variables. To ensure model robustness, accounting for 10% data incompleteness, the final calculated minimum total sample size was 300 patients. This study ultimately enrolled 343 eligible patients. Statistical power was verified (1–β > 85%), meeting the requirements for subsequent multivariable analysis and machine learning modeling.

Inclusion criteria: (1) Age 18–75 years; (2) Diagnosis of traumatic, unilateral, closed long bone (tibia, femur, humerus) diaphyseal or metaphyseal fracture; (3) Treatment with open reduction and internal fixation; (4) Availability of standardized serial follow-up data, including clinical review and imaging (X-ray and CT) data at least at 3, 6, and 9 months postoperatively; (5) Completeness and extractability of key clinical indicators (e.g., smoking history, blood glucose, nutritional status) and imaging parameters at baseline and during follow-up.

Exclusion criteria: (1) Pathological fracture or open fracture (Gustilo type III or above); (2) Comorbid severe lower extremity vascular disease, peripheral neuropathy, or chronic renal insufficiency known to significantly affect bone metabolism; (3) Deep infection after internal fixation requiring reoperation; (4) Removal of internal fixation for other reasons or receiving secondary bone grafting intervention during follow-up (before outcome determination); (5) Poor quality imaging data precluding accurate quantitative measurement; (6) Missing key information in clinical records.

All data were independently collected and entered into a dedicated database by two researchers blinded to the final outcomes. Clinical data were extracted from the electronic medical record system. Imaging data were retrieved from the Picture Archiving and Communication System and analyzed using specialized medical image processing software under blinded conditions.

### Data collection

Data for this study were sourced from the Department of Orthopedics’ electronic medical record system, Picture Archiving and Communication System, and postoperative follow-up outpatient databases. Two uniformly trained researchers, blinded to the final outcomes, independently collected and cross-checked the data to ensure accuracy.

Collected data encompassed the following four dimensions:

1. Demographics and baseline clinical characteristics: Age, sex, body mass index; Lifestyle habits (smoking history, defined as current smoking or quitting within 6 months prior to the fracture); Comorbidities (focusing on diabetes history, assessed by preoperative or early postoperative glycated hemoglobin levels for glycemic control); Nutritional status (preoperative serum albumin level); Primary injury mechanism (e.g., traffic accident, fall from height).
2. Injury and surgery-related characteristics: Fracture location (tibia/femur/humerus); Fracture type [classified according to the AO Foundation/Orthopaedic Trauma Association Fracture and Dislocation Classification (AO/OTA)]; Soft tissue injury severity (assessed by Gustilo classification for closed or open injuries); Time to surgery (interval from injury to surgery); Internal fixation method (e.g., intramedullary nailing, locking plate); Complexity of surgical intervention (graded based on factors like primary bone grafting or need for complex soft tissue reconstruction).
3. Postoperative recovery and follow-up indicators: Early postoperative systemic inflammatory response (quantified by peak C-reactive protein level in the first postoperative week); Patient compliance (assessed via outpatient review and questionnaires evaluating adherence to the recommended weight-bearing plan, calculated as the absolute deviation of “recommended weight-bearing percentage - patient-reported average weight-bearing percentage”); Time points for serial imaging evaluation (postoperative 1, 3, 6, and 9 months).
4. Quantitative imaging indicators: All patients underwent X-ray and CT scans of the affected limb at specified time points. Advanced imaging examinations including SPECT/CT, DCE-MRI, MRI and Doppler ultrasound were performed in 61.8% of the patients according to clinical needs, with a missing rate of 38.2% for advanced imaging parameters. The collected advanced imaging parameters included bone metabolic activity ratio based on SPECT/CT, microcirculatory perfusion parameters based on DCE-MRI, intramedullary edema volume based on MRI and ultrasound blood flow perfusion index. Based on CT images, specialized medical imaging software was used to quantitatively measure: Structural indicators — Maximum fracture gap width, volume of cystic change at the fracture site, relative bone density at the fracture ends (compared to adjacent normal bone tissue), three-dimensional callus volume (used to calculate callus volume growth rate); Standardized score — The Radiographic Union Scale for Tibial fractures (RUST) scoring system was used to assess healing progress. Some patients underwent advanced imaging based on clinical need, from which functional indicators were extracted: Bone metabolic activity ratio based on Single-Photon Emission Computed Tomography/Computed Tomography (SPECT/CT); Microcirculatory perfusion parameters based on dynamic contrast-enhanced magnetic resonance imaging (MRI); Intramedullary edema volume based on MRI; Blood flow perfusion index at the fracture site based on Doppler ultrasound.

All quantitative imaging parameters were measured with standardized processing and segmentation protocols to ensure accuracy and reproducibility. (1) Segmentation method: Semi-automated segmentation was used for callus volume and cystic change volume—two trained researchers manually delineated the region of interest (ROI) of the fracture site on axial, coronal and sagittal CT images, and the software automatically calculated the volume; microcirculatory perfusion parameters were obtained by automated quantitative analysis after manual ROI positioning on DCE-MRI images. (2) Inter-rater reliability assessment: 20% of the imaging data were randomly selected for independent measurement by two senior musculoskeletal radiologists, and the intraclass correlation coefficient (ICC) was used for evaluation. The ICC values of callus volume growth rate, cystic change volume and microcirculatory perfusion parameters were 0.92, 0.94 and 0.89, respectively, indicating excellent inter-rater consistency. (3) Predefined thresholds: Cystic changes at the fracture site were defined as hypodense lesions with CT value ≤20 HU and no enhancement in enhanced CT scanning; intramedullary edema was defined as hyperintense lesions in MRI T2WI-FS and the edema area exceeding 10% of the medullary cavity volume at the fracture segment, and the thresholds were uniformly applied to all patients. (4) Image processing pipeline: CT and MRI data were processed using Mimics 21.0 (Materialise, Belgium) and AW Server 5.6 (GE Healthcare, USA); CT reconstruction parameters were slice thickness 1 mm, reconstruction interval 0.5 mm, with standard soft tissue and bone algorithm; DCE-MRI perfusion parameters were calculated by the Tofts model with a temporal resolution of 5 s; Doppler ultrasound data were analyzed by the built-in software of Philips iU22 (Philips Healthcare, Netherlands) with a 7.5–10 MHz linear array probe.

### Outcome definition

Referring to international consensus on fracture healing and the definition standards in the Clinical Practice Guideline from the Orthopaedic Trauma Association (9, 10), and considering the biological time window for fracture healing along with objective imaging evidence, patients in this study were categorized into either the Union group or the Nonunion group. Group assignment was based on the following criteria:

Union group: Patients must meet all the following conditions: (1) Clinical Healing Criteria: The fracture site achieved painless clinical union during follow-up, manifesting as absence of local tenderness, no abnormal movement, ability for full weight-bearing, and satisfactory functional recovery; (2) Radiographic Healing Criteria: Serial X-ray and CT images showed blurring or disappearance of the fracture line with continuous callus bridging the fracture ends; (3) Time and Progression Criteria: The aforementioned signs of healing appeared within 9 months post-fracture surgery, and consecutive follow-up imaging (interval ≥3 months) demonstrated progressive or stable healing; (4) Quantitative Score Support: At the assessment time point, RUST score ≥10 (applicable to long bones).

Nonunion group: Patients meeting any of the following conditions were assigned to this group: (1) Time and Stagnation Criteria: At least 9 months post-fracture surgery, with serial imaging examinations (X-ray/CT) over a consecutive 3-month period showing no signs of any healing progression at the fracture site; (2) Typical Radiographic Evidence: Clear radiographic signs of nonunion, such as a persistently clear fracture line, sclerotic fracture ends, the presence of a gap, or pseudarthrosis formation; (3) Clinical and Radiographic Composite Evidence: Presence of clinical symptoms like pain and abnormal movement at the fracture site, concurrently meeting the imaging criteria in (1) or (2); (4) Quantitative Score Support: At the 9-month postoperative assessment time point, the RUST score persistently remained ≤7.

All radiographic assessments were independently performed by one senior orthopedic surgeon and one musculoskeletal radiologist under double-blind conditions (i.e., unaware of patients’ clinical data, laboratory results, and imaging reports from other time points). A pre-defined standardized protocol and measurement tools were used to evaluate, measure, and calculate RUST scores from the serial images. Efficacy determination (i.e., final group assignment) was independently made by these two assessors based on the composite criteria above. In case of disagreement, a third senior orthopedic expert from the research team arbitrated based on the original imaging data and pre-set criteria to reach a final consensus grouping.

All clinical data, imaging data, and assessment results were entered into a dedicated research database. Logical checks were performed to ensure the integrity, accuracy, and traceability of the data chain. The definitive diagnosis of union and nonunion was made at 9 months postoperatively in accordance with international consensus, while the prediction model for post-traumatic nonunion in this study is intended to be applied at 3 months postoperatively, and all predictive indicators in the model are derived from clinical and imaging data collected at 3 months postoperatively (callus volume growth rate is calculated from 1 and 3 months postoperative CT data), realizing early risk assessment of fracture healing.

### Statistical analysis

Data analysis was performed using SPSS 26.0, R 4.3.1, and Python 3.9.12 software. Measurement data conforming to a normal distribution are presented as mean ± standard deviation (x̄ ± s), and intergroup comparisons were conducted using independent samples t-tests. Count data are presented as number (percentage) [*n*(%)], and intergroup comparisons were made using the *χ*^2^ test or Fisher’s exact test.

The dataset was split 7:3 into training and validation sets, and all feature selection steps were strictly performed within the training set to avoid information leakage: first, univariate analysis was performed to screen variables with *p* < 0.05; subsequently, LASSO regression was used for variable compression; finally, multivariable logistic regression was employed to identify independent influencing factors. Sample size calculation followed prediction model guidelines and adhered to the “at least 10 events per variable (EPV)” rule to ensure model stability (Variance Inflation Factor, VIF < 5 for all variables). A total of 32 initial candidate predictors (12 clinical indicators and 20 imaging indicators including conventional and advanced imaging parameters) were included in the feature selection; 10 predictors were retained after LASSO regression, and 6 statistically significant independent predictors were finally identified by multivariable logistic regression.

Based on the results of the multivariable analysis, machine learning models including Random Forest, Gradient Boosting Machine, and K-Nearest curve were constructed using Python 3.9.12 and the scikit-learn library. 10-fold cross-validation was used within the training set for hyperparameter tuning of all machine learning models: for GBM, the learning rate (0.01–0.1), maximum tree depth (3–10), number of estimators (100–500) and subsample ratio (0.7–1.0) were optimized; for KNN, the K value (3–15) and distance metric (Euclidean, Manhattan) were optimized; for Random Forest, the number of estimators (100–500) and maximum tree depth (3–10) were optimized. The optimal hyperparameter combination was selected according to the highest AUC value of the cross-validation internal validation set, and a single final evaluation of the model performance was conducted on the held-out 30% independent validation set. A multi-dimensional overfitting control strategy was adopted for the GBM model during training to ensure its robustness: (1) Limiting the maximum tree depth to 3–5 to avoid overfitting caused by complex tree structures; (2) Setting a low learning rate of 0.01–0.05 and matching with an appropriate number of estimators to realize slow and stable learning; (3) Adopting subsampling (0.7–0.9) and column sampling (0.8–0.9) to reduce the correlation between decision trees; (4) Enabling early stopping with a patience of 20 to stop training when the model performance no longer improves; (5) Adding L2 regularization (alpha = 0.1–0.5) to limit the parameter weight. In addition, hold-out validation and calibration curve analysis were used to further verify the generalization ability of the model and avoid overfitting. Model predictive performance was evaluated using the area under the receiver operating characteristic curve (AUC). Calibration was assessed via calibration curves and the Hosmer-Lemeshow test. Clinical utility was evaluated using decision curve analysis. Concurrently, a nomogram prediction model was built using the “rms” package in R software and internally validated via the Bootstrap method. Additionally, SHAP values were calculated using Python’s “shap” library to assess model interpretability.

## Results

### Comparison of general characteristics between the training and validation sets

A total of 343 patients with post-traumatic fractures meeting the inclusion and exclusion criteria were enrolled. They were randomly divided into a training set (*n* = 240) and a validation set (*n* = 103) in a 7:3 ratio. Comparisons of baseline characteristics between the training and validation sets showed no statistically significant differences (*p* > 0.05) in terms of age, smoking history, preoperative nutritional status (serum albumin), glycemic control level, injury severity, complexity of surgical intervention, postoperative inflammatory response, treatment compliance (weight-bearing deviation), and various imaging indicators. These imaging indicators included structural parameters (maximum fracture gap width, volume of cystic change at fracture site, relative bone density, signs of implant failure), healing progression indicators (callus volume growth rate, RUST score), and functional imaging parameters (bone metabolic activity ratio, microcirculatory perfusion, intramedullary edema volume, ultrasound blood flow perfusion index). This indicates a balanced data partition between the training and validation sets, with good comparability in baseline characteristics, laying a foundation for subsequent prediction model development and validation (Table 1).

**Table 1:** Comparison of general characteristics between the training and validation sets.

Variables	Training set (*n* = 240)	Validation set (*n* = 103)	*t/χ^2^*	*p*
Age (years)	51.23 ± 15.67	52.89 ± 14.32	0.673	0.502
Sex				
Male	150 (62.5%)	65 (63.1%)	0.013	0.914
Female	90 (37.5%)	38 (36.9%)		
Smoking history				
Yes	68 (28.33%)	31 (30.10%)	0.124	0.724
No	172 (71.67%)	72 (69.90%)		
Alcohol use history				
Yes	52 (21.7%)	24 (23.3%)	0.123	0.726
No	188 (78.3%)	79 (76.7%)		
Osteoporosis				
Yes	38 (15.8%)	18 (17.5%)	0.154	0.695
No	202 (84.2%)	85 (82.5%)		
Glucocorticoid use history				
Yes	12 (5.0%)	5 (4.9%)	0.012	0.968
No	228 (95.0%)	98 (95.1%)		
Preoperative serum albumin (g/L)	38.45 ± 4.56	37.89 ± 4.78	0.759	0.448
Glycemic control level (%)	6.12 ± 1.45	6.08 ± 1.51	0.171	0.865
Injury severity index				
1	45 (18.75%)	20 (19.42%)	0.020	0.999
2	98 (40.83%)	42 (40.78%)		
3	72 (30.00%)	31 (30.10%)		
4	25 (10.42%)	10 (9.71%)		
Surgical intervention complexity				
1	80 (33.33%)	35 (33.98%)	0.037	0.982
2	120 (50.00%)	52 (50.49%)		
3	40 (16.67%)	16 (15.53%)		
Peak postoperative inflammation (mg/L)	35.67 ± 18.24	34.12 ± 17.85	0.544	0.587
Weight-bearing compliance deviation (%)	25.43 ± 12.56	26.78 ± 13.21	0.680	0.497
Maximum fracture gap width (mm)	3.45 ± 1.89	3.67 ± 1.95	0.739	0.461
Cystic change volume at fracture site (cm^3^)	2.34 ± 1.45	2.56 ± 1.52	0.960	0.338
Relative bone mineral density at fracture site	0.85 ± 0.23	0.83 ± 0.22	0.559	0.577
Callus volume growth rate (%)	15.34 ± 8.90	14.56 ± 9.21	0.556	0.579
Signs of internal fixation failure score				
0	180 (75.00%)	78 (75.73%)	0.360	0.719
1	40 (16.67%)	17 (16.50%)		
2	15 (6.25%)	6 (5.83%)		
3	5 (2.08%)	2 (1.94%)		
RUST score (points)	7.89 ± 2.34	7.65 ± 2.41	0.651	0.516
Bone metabolic activity ratio	1.98 ± 0.76	2.05 ± 0.81	0.581	0.562
Microcirculatory perfusion parameter	0.45 ± 0.18	0.42 ± 0.17	1.073	0.284
Intramedullary edema volume (cm^3^)	12.34 ± 5.67	11.89 ± 5.34	0.511	0.610
Ultrasound blood flow perfusion index (%)	8.56 ± 3.45	8.23 ± 3.67	0.604	0.547

### Univariate analysis of factors influencing post-traumatic nonunion in the training cohort

In the training cohort of 240 patients, 191 (79.6%) achieved bone union, while 49 (20.4%) developed nonunion. Univariate analysis revealed that the differences in age, Injury Severity Index, maximum fracture gap width, cystic change volume at the fracture site, callus volume growth rate, standardized bone healing score (RUST) between the union and nonunion groups were statistically significant (*p* < 0.05). In contrast, smoking history, nutritional and glycemic status, surgical complexity, inflammation levels, treatment compliance, microcirculatory perfusion parameters and other imaging indicators showed no statistically significant differences between the two groups (*p* > 0.05). These findings suggest that initial injury severity, structural conditions at the fracture site, callus growth dynamics, and local microcirculatory status may be key factors influencing fracture healing outcomes (Table 2).

**Table 2:** Univariate analysis of factors influencing post-traumatic nonunion in the training cohort.

Variables	Union group (*n* = 191)	Nonunion group (*n* = 49)	*t/χ^2^*	*p*
Age (years)	47.08 ± 10.89	55.45 ± 14.23	2.956	0.003
Sex				
Male	118 (61.8%)	32 (65.3%)	0.105	0.746
Female	73 (38.2%)	17 (34.7%)		
Smoking history				
Yes	47(24.6%)	18(36.7%)	2.904	0.088
No	144(75.4%)	31(63.3%)		
Alcohol use history				
Yes	38 (19.9%)	10 (20.4%)	0.007	0.934
No	153 (80.1%)	39 (79.6%)		
Osteoporosis				
Yes	30 (15.7%)	8 (16.3%)	0.013	0.910
No	161 (84.3%)	41 (83.7%)		
Glucocorticoid use history				
Yes	9 (4.7%)	3 (6.1%)	0.178	0.673
No	182 (95.3%)	46 (93.9%)		
Preoperative serum albumin (g/L)	38.78 ± 4.67	37.42 ± 4.23	1.852	0.065
Glycemic control level (%)	6.05 ± 1.38	6.45 ± 1.67	1.731	0.085
Injury severity index				
1	45 (23.6%)	6 (12.2%)	9.212	0.027
2	92 (48.2%)	19 (38.8%)		
3	45 (23.6%)	18 (36.7%)		
4	9 (4.7%)	6 (12.2%)		
Surgical intervention complexity				
1	74 (38.7%)	16 (32.7%)	1.111	0.267
2	98 (51.3%)	22 (44.9%)		
3	19 (9.9%)	11 (22.4%)		
Peak postoperative inflammation (mg/L)	34.78 ± 17.89	38.90 ± 19.12	1.418	0.158
Weight-bearing compliance deviation (%)	24.89 ± 12.23	27.34 ± 13.45	1.225	0.222
Maximum fracture gap width (mm)	2.98 ± 1.45	5.12 ± 1.89	8.628	0.001
Cystic change volume at fracture site (cm^3^)	1.99 ± 0.98	3.53 ± 2.01	7.647	0.001
Relative bone mineral density at fracture site	0.86 ± 0.23	0.81 ± 0.21	1.381	0.169
Callus volume growth rate (%)	17.23 ± 8.12	8.45 ± 5.67	7.131	0.001
Signs of internal fixation failure score				
0	148 (77.5%)	34 (69.4%)	0.477	0.490
1	28 (14.7%)	10 (20.4%)		
2	11 (5.8%)	4 (8.2%)		
3	4 (2.1%)	1 (2.0%)		
RUST score (points)	8.45 ± 1.89	6.78 ± 1.23	5.870	0.001
Bone metabolic activity ratio	2.01 ± 0.78	1.85 ± 0.71	1.304	0.194
Microcirculatory perfusion parameter	0.48 ± 0.15	0.43 ± 0.12	1.592	0.112
Intramedullary edema volume (cm^3^)	12.01 ± 5.56	13.45 ± 5.89	1.598	0.111
Ultrasound blood flow perfusion index (%)	8.78 ± 3.56	7.89 ± 3.12	1.599	0.111

### Multivariate logistic regression analysis of factors influencing post-traumatic nonunion

**Figure 1:**
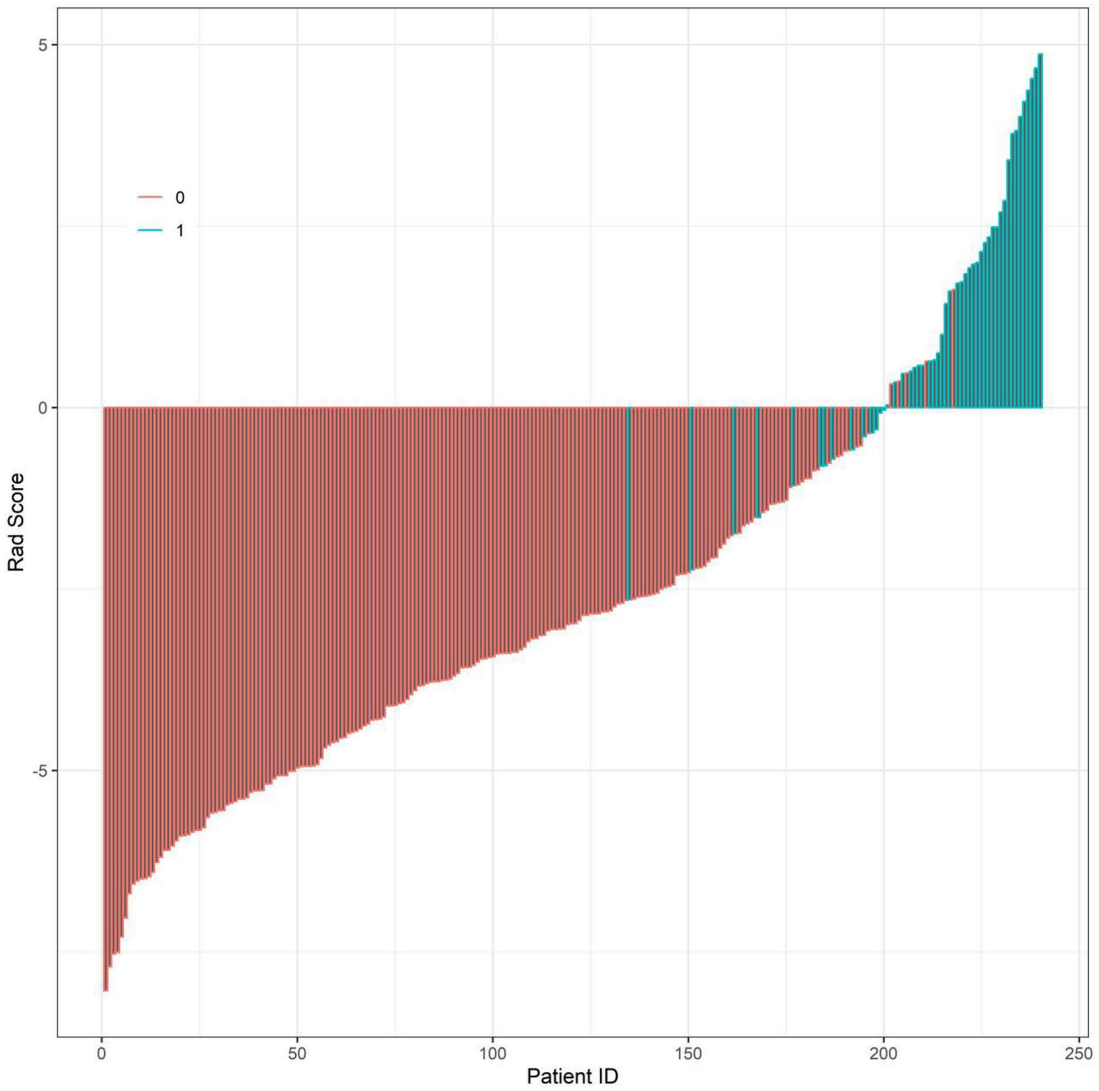
Lasso plot.

Using post-traumatic nonunion as the dependent variable (1 = nonunion group, 0 = union group), the six indicators identified as statistically significant in the univariate analysis (Injury Severity Index, maximum fracture gap width, cystic change volume at the fracture site, callus volume growth rate, RUST score, microcirculatory perfusion parameters) were included as candidate predictor variables in a LASSO regression model for feature selection (Supplementary Table 1). The optimal regularization parameter *λ* was determined using 10-fold cross-validation and the minimum error (λ-min) criterion (Figure 1). Under this criterion, the LASSO regression ultimately selected and retained all six candidate variables, indicating that each contributes importantly to the model. These six LASSO-selected variables were then incorporated into a multivariate binary logistic regression model to further clarify their independent predictive value and calculate their effect sizes. The multivariate logistic regression analysis results (Table 3) showed that the Injury Severity Index, maximum fracture gap width, and cystic change volume at the fracture site were independent risk factors for post-traumatic nonunion (*p* < 0.05). Conversely, the callus volume growth rate and RUST score were independent protective factors against nonunion (*p* < 0.05).

**Table 3:** Multivariate logistic regression analysis of factors influencing post-traumatic nonunion.

Indicator	*β*	SE	Wald	*p*	OR	95% CI
Age	0.080	0.027	9.098	0.003	1.084	1.029 ~ 1.142
Injury severity index	1.131	0.258	19.151	0.001	3.099	1.867 ~ 5.143
Maximum fracture gap width	0.939	0.227	17.120	0.001	2.556	1.639 ~ 3.988
Cystic change volume at fracture site	1.031	0.219	22.214	0.001	2.804	1.826 ~ 4.305
Callus volume growth rate	−0.157	0.047	13.326	0.001	0.855	0.779 ~ 0.937
RUST score	−0.868	0.238	13.326	0.001	0.420	0.263 ~ 0.669

### Machine learning model performance evaluation

**Figure 2:**
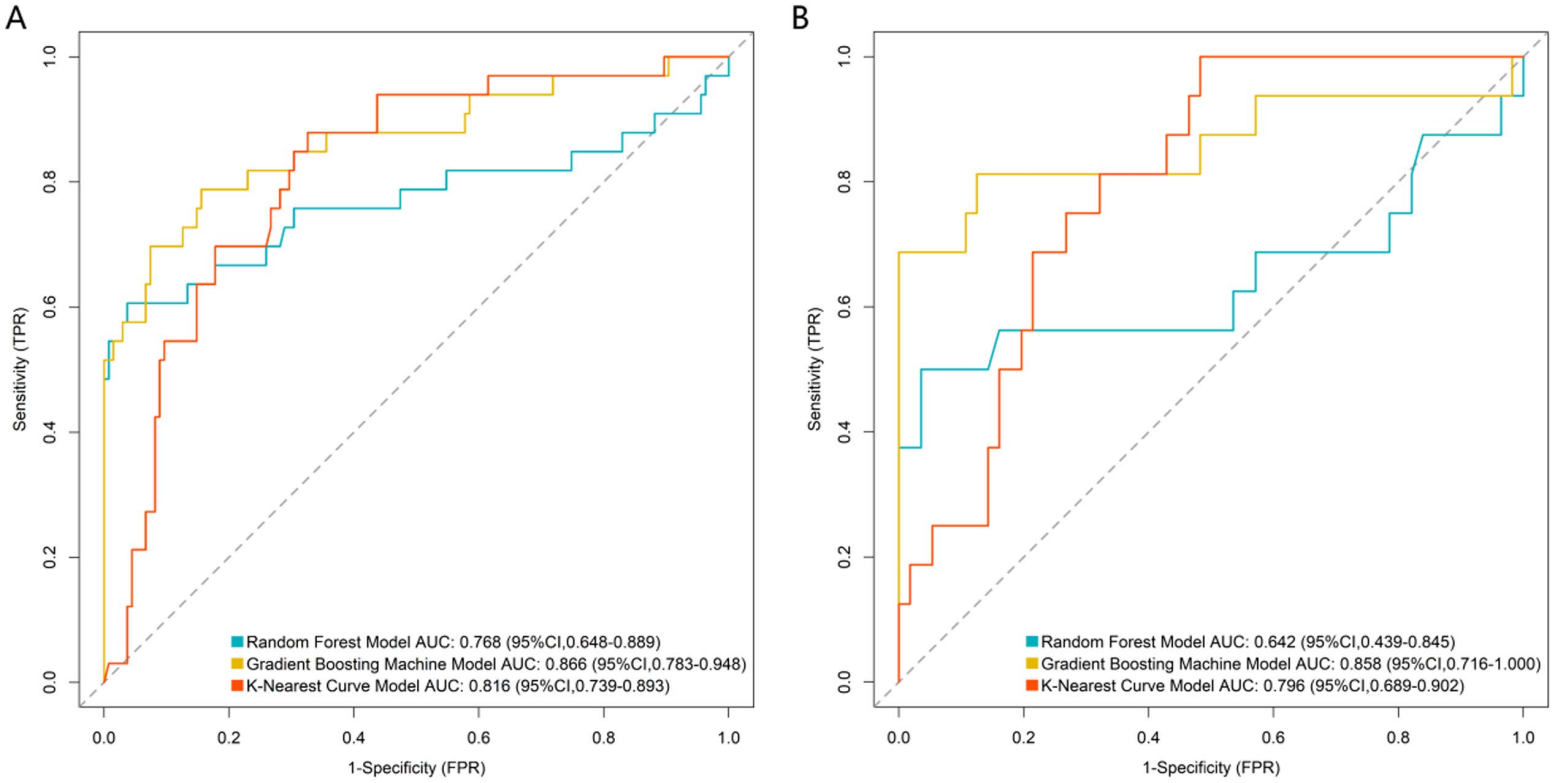
ROC curve analysis of the prediction model in the training **(A)** and validation **(B)** sets.

**Figure 3:**
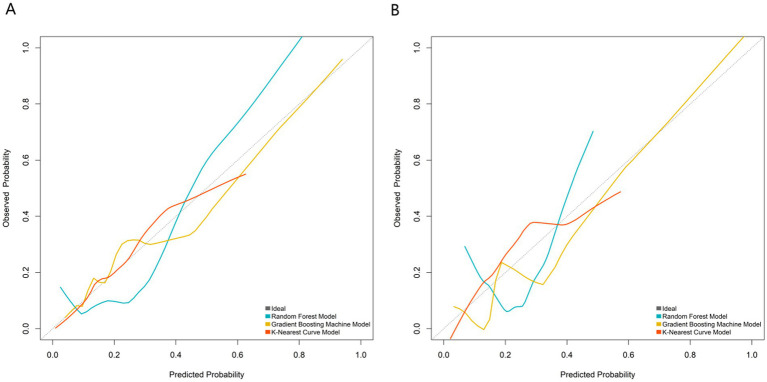
Curve analysis of the prediction model in the training **(A)** and validation **(B)** sets.

**Figure 4:**
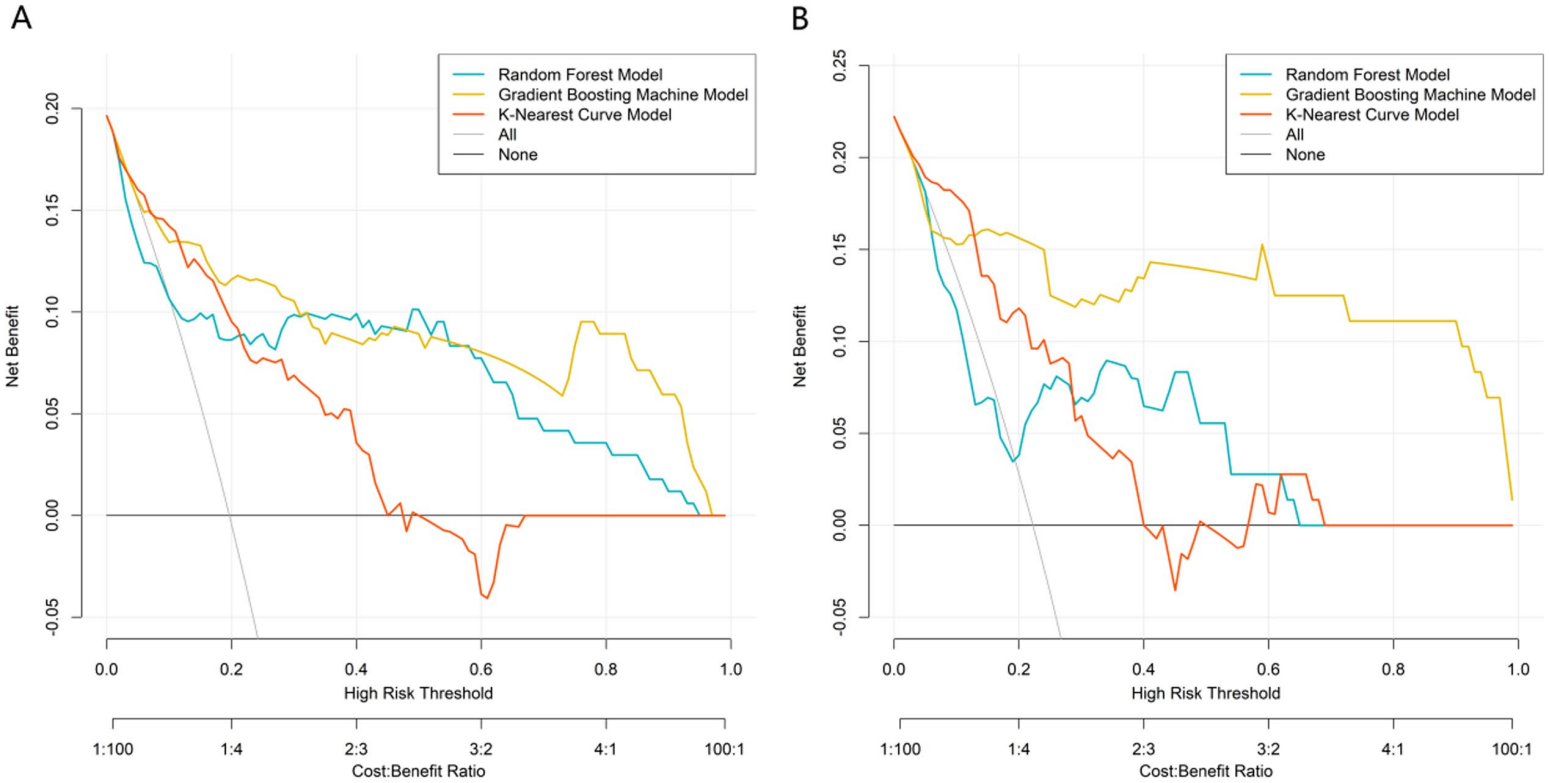
Clinical decision curve analysis of the prediction model in the training **(A)** and validation **(B)** sets.

All predictors of the machine learning models were based on clinical and imaging data at 3 months postoperatively, and the models were constructed to predict the risk of nonunion at 9 months postoperatively, which is an early prediction model for clinical application at 3 months postoperatively. Based on the independent predictor variables, three machine learning models (Random Forest, Gradient Boosting Machine, K-Nearest Curve) were constructed to evaluate predictive performance. For the training set (Figure 2A): the Gradient Boosting Machine model achieved the highest AUC of 0.866 (95% CI: 0.783–0.948), followed by the K-Nearest Curve model (AUC: 0.816; 95% CI: 0.739–0.893) and the Random Forest model (AUC: 0.768; 95% CI: 0.648–0.889). For the validation set (Figure 2B): the Gradient Boosting Machine model still maintained a high AUC of 0.858 (95% CI: 0.716–1.000), outperforming the K-Nearest Curve model (AUC: 0.796; 95% CI: 0.689–0.902) and the Random Forest model (AUC: 0.642; 95% CI: 0.439–0.845). The EPV of the multivariable logistic regression model in this study was about 8.2 (49 nonunion events/6 independent predictors), and a multi-dimensional overfitting control strategy was specially adopted for the GBM model to ensure its robustness, which was not only based on the EPV argument of logistic regression. Thus, the Gradient Boosting Machine model was identified as the optimal predictor, with stable and superior discriminative ability for post-traumatic nonunion. Further calibration curve analysis (Figure 3) showed that the Gradient Boosting Machine model’s predicted probabilities closely aligned with the observed actual risk (its curve tracked the diagonal “Ideal” line most closely) in both the training (Figure 3A) and validation (Figure 3B) sets. This indicates excellent predictive calibration and reliable accuracy of the model’s risk estimates. Additionally, decision curve analysis (Figure 4) demonstrated that across most high-risk thresholds, the net benefit curve of the Gradient Boosting Machine model consistently exceeded the extreme strategies of “intervene on all patients” or “intervene on none” in both the training (Figure 4A) and validation (Figure 4B) sets. This clinical utility was particularly prominent in the high-risk range, suggesting that using this model to identify high-risk nonunion patients (for targeted interventions like enhanced monitoring or early bone grafting) yields greater clinical net benefit. In summary, the Gradient Boosting Machine model—built on key clinical/imaging indicators—exhibits strong predictive accuracy, calibration, and clinical applicability. It provides objective quantitative support for individualized healing risk assessment and clinical decision-making in post-traumatic fracture patients, with high translational potential.

### Interpretability assessment of model predictions

Based on the five independent predictors (Injury Severity Index, maximum fracture gap width, cystic change volume at the fracture site, callus volume growth rate, RUST score) identified by multivariate logistic regression, this study developed a nomogram model (Supplementary Figure 1A) for predicting post-traumatic nonunion risk. This nomogram visually delineates each variable’s independent contribution to nonunion risk: X1 (Injury Severity Index), X2 (maximum fracture gap width), and X3 (cystic change volume) act as risk factors (higher values correlate with higher point scores), while X4 (callus volume growth rate) and X5 (RUST score) serve as protective factors (higher values link to lower point scores). To quantify individualized risk, clinicians can map a patient’s specific values for each variable to the corresponding “Points” axis, sum these scores to get the “Total points,” and then read the matching nonunion probability from the bottom axis (e.g., the total score in the nomogram aligns with a risk of ~0.00049). To clarify the model’s decision logic, SHapley Additive exPlanations (SHAP) analysis was conducted (Supplementary Figure 1B): the SHAP summary plot quantifies each predictor’s contribution direction and magnitude to the model output. The order of feature influence (from strongest to weakest) is X2, X3, X4, X5, X1. Notably, higher feature values (orange points) of X2 and X3 correspond to larger positive SHAP values (elevating nonunion risk), while higher feature values of X4 and X5 align with smaller (or negative) SHAP values (reducing nonunion risk). These results align with multivariate logistic regression conclusions, confirming that fracture site structural conditions (gap, cystic changes) and healing activity dynamics (callus growth, RUST score) at 3 months postoperatively are the core features driving the model’s predictions, which further supports the clinical value of early prediction at 3 months postoperatively. By integrating the clinically accessible nomogram (for practical risk quantification) and mechanism-revealing SHAP analysis (for transparent decision logic), this study not only provides a user-friendly tool for personalized nonunion risk assessment but also elucidates the model’s decision basis via feature contributions—enhancing the transparency, interpretability, and clinical credibility of predictions, and offering orthopedic surgeons solid guidance for early high-risk patient identification and targeted intervention planning. To translate the statistical SHAP analysis results into actionable clinical decision-making, the practical clinical significance of different SHAP values of core predictors and corresponding intervention strategies are clearly explained with specific threshold values based on the actual data of this study. The SHAP analysis revealed the feature importance ranking (from highest to lowest): maximum fracture gap width, cystic change volume, callus volume growth rate, RUST score, Injury Severity Index. A high positive SHAP value of the maximum fracture gap width indicates a larger fracture gap and a greater contribution to increased nonunion risk; a low negative SHAP value of the RUST score indicates a lower score and a greater contribution to increased nonunion risk. Specific clinical risk thresholds and intervention suggestions are as follows: (1) Extremely high-risk patients: Patients with a maximum fracture gap width >5 mm (top 25% of the study population, high SHAP value) and a RUST score <6 points (bottom 25% of the study population, low SHAP value) at 3 months postoperatively have a predicted nonunion risk >85%; patients with a cystic change volume >4 cm^3^ (high SHAP value) and a callus volume growth rate <5% (low SHAP value) have a predicted nonunion risk >80%. For these patients, aggressive early interventions are recommended, including enhanced follow-up (once a month), personalized weight-bearing restriction, administration of anti-osteoporosis and vascular improvement drugs, and early surgical bone grafting if the healing trend is still poor after 1 month of conservative intervention. (2) Moderate-risk patients: Patients with a maximum fracture gap width of 3–5 mm, a RUST score of 6–8 points, a cystic change volume of 2–4 cm^3^ or a callus volume growth rate of 5%–10% have a predicted nonunion risk of 30%–80%. For these patients, strengthened monitoring and targeted rehabilitation are recommended, including follow-up every 6 weeks, regular re-evaluation of imaging indicators, and adjustment of the rehabilitation plan according to the callus growth status. (3) Low-risk patients: Patients with a maximum fracture gap width <2 mm (low SHAP value), a RUST score >9 points (high SHAP value), a cystic change volume <2 cm^3^ or a callus volume growth rate >10% have a predicted nonunion risk <5%. For these patients, routine clinical follow-up and standard rehabilitation guidance are sufficient, without additional intervention measures. This clinical translation scheme fully links the SHAP statistical results with specific clinical decision-making, making the model’s predictive output more intuitive and actionable for orthopedic clinicians, and providing a clear reference for individualized risk stratification and intervention planning.

## Discussion

Post-traumatic nonunion represents a challenging clinical problem in orthopedics. Its occurrence is influenced by multiple factors, and there is currently a lack of objective, quantitative tools for the early and accurate identification of high-risk patients. This study innovatively integrated key clinical risk indicators (e.g., injury severity) with multi-modal quantitative imaging features (conventional X-ray/CT structural and dynamic parameters, and advanced imaging functional parameters for initial screening) to successfully develop and validate a comprehensive model for predicting post-traumatic nonunion, in which advanced imaging parameters were excluded during feature selection due to low contribution and no statistical significance. The model demonstrated excellent discriminative ability in the validation cohort (Gradient Boosting Machine model AUC = 0.866). The robustness of predictors and model performance optimization were ensured through systematic comparisons involving LASSO regression, multivariate logistic regression, and various machine learning algorithms. More importantly, we conducted an in-depth interpretability analysis of the model using a nomogram and SHAP analysis. This not only quantified the relative importance of each predictor but also clarified its quantitative relationship with nonunion risk, providing a new perspective and a decision-making basis for early clinical risk warning and personalized intervention.

The five core predictors ultimately identified in this study precisely capture three critical dimensions influencing fracture healing outcomes: initial conditions, healing activity, and structural outcome. Among them, the maximum fracture gap width and the volume of cystic change at the fracture site were proven to be the most influential predictors. An immediately post-operative wide fracture gap (>2–3 mm) is a direct mechanical impediment to bone conduction and mechanical stability. Its presence not only hinders the migration of nascent blood vessels and osteoprogenitor cells but can also lead to excessive stress on internal fixation, inducing micromotion and mechanical instability, thereby disrupting the hematoma organization and soft callus formation stages in early healing (11, 12). Cystic change at the fracture site is a typical imaging hallmark of biological inactivation and predominant bone resorption during the healing process. Its formation is closely associated with local inadequate blood supply, dysregulated inflammatory response, and relatively hyperactive osteoclast activity. It reflects an imbalance between pro-healing signals (e.g., BMPs, VEGF) and inhibitory signals (e.g., inflammatory cytokines, mechanical instability) within the fracture microenvironment, ultimately leading to the formation of bone resorption lacunae instead of callus mineralization (13, 14). The high contribution of these two structural indicators underscores the foundational role of precise imaging assessment in prediction.

It is particularly noteworthy that the callus volume growth rate and the RUST score were identified as key independent protective factors, serving as dynamic and semi-quantitative healing indicators. The callus volume growth rate directly quantifies the rate of mesenchymal stem cell differentiation into osteoblasts and extracellular matrix mineralization, representing the most intuitive kinetic reflection of the “osteogenic potential” of bone healing (15, 16). Its slow growth or stagnation often suggests insufficient local stem cell recruitment, impaired osteogenic differentiation, or inadequate blood supply to support robust metabolic demands. The RUST score provides a standardized, reproducible comprehensive assessment of healing status by evaluating callus bridging, remodeling, and cortical continuity (17). Their significant protective effects confirm that the biological activity of the healing process is a more powerful prognostic determinant than the initial injury conditions. This implies that dynamic monitoring of healing progression during clinical follow-up holds greater predictive value than a single assessment.

Furthermore, the injury severity index, as a clinical risk stratification indicator, had its predictive value confirmed within our model. High-energy trauma and open fractures are typically associated with more severe soft tissue damage and compromised blood supply. This not only directly disrupts the critical peri-fracture vascular network (e.g., periosteal and intramedullary vessels) but can also trigger a intense early inflammatory response. If this response is excessive or becomes chronic, it may lead to fibrous tissue proliferation instead of bone formation (18, 19). However, its lower contribution in the SHAP analysis compared to the core imaging indicators suggests that within the comprehensive model, direct evidence reflecting the current healing state (imaging indicators) possesses stronger predictive power than indirect evidence describing the initial risk (clinical indicators). This also indicates that even with a severe initial injury, favorable healing is still possible if a conducive local biological environment (e.g., stable fixation, infection control, promoted vascularity) can be created through surgery and post-operative management. Clinical feasibility and implementation barriers are key factors affecting the translational potential of the prediction model, and the accessibility of imaging modalities and the applicability of simple surrogate markers in resource-limited settings are fully discussed here. DCE-MRI and SPECT/CT are currently mainly available in tertiary hospitals in China with a penetration rate of 60%–70%, while they are rarely equipped in secondary and primary hospitals due to high costs; Doppler ultrasound has a high accessibility (>90%) in hospitals at all levels, but its quantitative perfusion analysis is not a routine examination. Notably, the core predictors of the final model (maximum fracture gap width, cystic change volume, callus volume growth rate, RUST score) are all derived from basic CT and X-ray data, which are widely available in hospitals at all levels. Our study confirmed that these simple surrogate markers alone can achieve high predictive accuracy (AUC = 0.858 in the validation set), indicating that advanced imaging parameters are not necessary for routine clinical application, and simple markers are sufficient for nonunion risk prediction in resource-limited settings. To further improve the clinical translational potential, we have developed a simplified nomogram model based on three widely available X-ray/CT indicators (maximum fracture gap width, RUST score, callus volume growth rate) for secondary and primary hospitals. The simplified model retains the core predictive ability of the original model (AUC = 0.823 in internal validation) and is more user-friendly—clinicians can complete individualized nonunion risk assessment only by measuring the three indicators from routine X-ray/CT images without advanced imaging equipment or complex machine learning analysis software. In future research, we plan to extract radiomic features from routine X-ray images using deep learning technology to further optimize the simplified model, aiming to balance predictive accuracy and clinical applicability, and maximize the translational value of the model in hospitals at all levels.

The core methodological strength of this study lies in the deep integration of clinical and imaging data and the application of advanced machine learning and interpretability techniques. The strategy of initially screening high-dimensional features via LASSO regression, followed by identifying independent risk ratios using logistic regression, and finally constructing a high-performance prediction model with gradient boosting, balanced statistical rigor with predictive accuracy (20). The application of SHAP analysis is a highlight of this research. It demystifies the model’s “black box” decision-making process, objectively displaying the positive or negative contribution of each feature to individual prediction outcomes. This significantly enhances the model’s credibility and acceptability among clinicians, aligning with the development trend of explainable artificial intelligence in medical applications (21).

This study has several limitations. First, it is a single-center, retrospective study. Although rigorous internal validation was performed, the model’s external generalizability requires further verification through multi-center, prospective cohorts. Second, the acquisition of certain advanced imaging parameters (e.g., perfusion parameters) depends on specific equipment, potentially limiting the model’s adoption in primary care hospitals. Future work could explore developing simplified yet highly predictive models by extracting deeper features from more widely available X-ray or CT images using radiomics technology (22). Finally, the model did not incorporate some potentially relevant systemic biomarkers (e.g., serum Procollagen Type I N-Terminal Propeptide, C-terminal Telopeptide of Type I Collagen for bone metabolism, or Interleukin-6, Tumor Necrosis Factor-alpha for systemic inflammation). These biomarkers might reflect the patient’s systemic bone metabolic status and inflammatory load. Future research could attempt to integrate multi-omics data, such as genomics and proteomics, to build a more comprehensive prediction framework (23). “Revised to”: This study has several limitations. First, it is a single-center, retrospective study, which has potential biases affecting the model’s generalizability: selection bias exists as the included patients are all from a single tertiary hospital with a relatively high proportion of severe trauma patients, which may overestimate the contribution of the Injury Severity Index to the model; the high consistency of surgical techniques (unified operation specifications for intramedullary nailing and locking plate) and imaging protocols (1 mm CT slice thickness, standardized X-ray shooting position) in the single center ensures the accuracy of predictor measurement, but the differences in surgical techniques, imaging equipment and protocols in other medical institutions may lead to deviations in predictor values such as callus volume growth rate; the local standardized treatment practices (e.g., postoperative anti-inflammation and rehabilitation guidance) may make the model more applicable to populations with similar treatment plans, and its adaptability in areas with different treatment practices needs to be verified. Although rigorous internal validation was performed, the model’s external generalizability must be further verified through multi-center, prospective cohort studies, which is the necessary and key next step for the real-world clinical application of the model, and our research team has planned to launch multi-center collaborative research for external validation. Second, the acquisition of certain advanced imaging parameters (e.g., perfusion parameters) depends on specific equipment, potentially limiting the model’s adoption in primary care hospitals. Future work could explore developing simplified yet highly predictive models by extracting deeper features from more widely available X-ray or CT images using radiomics technology (22). Finally, the model did not incorporate some potentially relevant systemic biomarkers (e.g., serum Procollagen Type I N-Terminal Propeptide, C-terminal Telopeptide of Type I Collagen for bone metabolism, or Interleukin-6, Tumor Necrosis Factor-alpha for systemic inflammation). These biomarkers might reflect the patient’s systemic bone metabolic status and inflammatory load. Future research could attempt to integrate multi-omics data, such as genomics and proteomics, to build a more comprehensive prediction framework (23). In conclusion, this study successfully developed and validated a comprehensive prediction model for post-traumatic nonunion by integrating clinical and multi-modal imaging indicators. The model not only exhibits excellent predictive performance but also, through interpretability analysis, deeply reveals that “initial structural conditions” and “healing process activity” are the core logic driving the predictions. The results emphasize the importance of combining static injury assessment with dynamic healing monitoring for accurate prognostic judgment. As a promising clinical decision-support tool, this model establishes a solid foundation for achieving personalized risk management in fracture patients, optimizing intervention timing (e.g., early bone grafting), and rationally allocating medical resources. Future efforts should focus on prospective external validation and dedicated development of simplified versions applicable across different levels of healthcare resources to maximize its clinical translational value.

## Data Availability

The raw data supporting the conclusions of this article will be made available by the authors, without undue reservation.

## References

[1] Bevoni R Artioli E Di Ponte M Caravelli S Mosca M. Personalized surgical approach for nonunion of the second metatarsal fracture and post-traumatic metatarsalgia: a case report and literature review. J Pers Med. (2025) 15:174. doi: 10.3390/jpm15050174, 40423046 PMC12113075

[2] Gabig AM Malmquist JA Brady CI Dutta AK. A novel hemiarthroplasty design for treatment of post-traumatic elbow ankylosis and distal humerus nonunion: a case report. JBJS Case Connect. (2022) 12:e22.00393. doi: 10.2106/jbjs.Cc.22.00393 36820842

[3] Gausden EB Tibbo ME Perry KI Berry DJ Yuan BJ Abdel MP. Outcomes of Vancouver C periprosthetic femur fractures. J Arthroplast. (2021) 36:3601–7. doi: 10.1016/j.arth.2021.05.033, 34119395

[4] Bhowmick K Jepegnanam TS Inja DB Karuppusami R Nithyananth M. The outcomes of surgical treatment for lateral Hoffa fracture nonunions. Arch Orthop Trauma Surg. (2023) 143:2509–17. doi: 10.1007/s00402-022-04503-4 35723709

[5] Chowdary AR Ravi V Wukich DK Sambandam S. Outcomes of surgically treated pilon fractures: a comparison of patients with and without diabetes. J Orthop Trauma. (2023) 37:650–7. doi: 10.1097/bot.0000000000002701, 37797331

[6] Punnoose DJ N N A NS Kandathil JC Theruvil B. Total hip arthroplasty in post-traumatic acetabular nonunion with symphysis pubis diastasis: a case report. JBJS Case Connect. (2021) 11:e20.01028. doi: 10.2106/jbjs.Cc.20.01028 34252069

[7] Li C Yang Z Yang P Li Z Wang T Xing B . Association between post-trauma platelet-lymphocyte ratio and nonunion in patients with extremity fractures: a multicenter retrospective cohort study. Int J Surg. (2025) 111:8943–52. doi: 10.1097/js9.0000000000003265 40844879 PMC12695322

[8] Nauth A Haller J Augat P Anderson DD McKee MD Shearer D . Distal femur fractures: basic science and international perspectives. OTA Int. (2024) 7:e320. doi: 10.1097/oi9.0000000000000320, 38487402 PMC10936154

[9] Calori GM Phillips M Jeetle S Tagliabue L Giannoudis PV. Classification of non-union: need for a new scoring system? Injury. (2008) 39:S59–63. doi: 10.1016/s0020-1383(08)70016-0 18804575

[10] Krappinger D Wolf B Dammerer D Thaler M Schwendinger P Lindtner RA. Risk factors for nonunion after intramedullary nailing of subtrochanteric femoral fractures. Arch Orthop Trauma Surg. (2019) 139:769–77. doi: 10.1007/s00402-019-03131-9, 30729990 PMC6514068

[11] Poff C Kunkle B Li X Friedman RJ Eichinger JK. Assessing the hospital volume-outcome relationship in total elbow arthroplasty. J Shoulder Elb Surg. (2022) 31:367–74. doi: 10.1016/j.jse.2021.08.025, 34592413

[12] Hadjiargyrou M Salichos L Kloen P. Identification of the mirnaome in human fracture callus and nonunion tissues. J Orthop Transl. (2023) 39:113–23. doi: 10.1016/j.jot.2023.01.005, 36909863 PMC9996375

[13] Nahm NJ Conway JD. Resorbable polylactide membrane for the treatment of segmental bone defects. Injury. (2022) 53:376–80. doi: 10.1016/j.injury.2021.11.024, 34852920

[14] Borzunov DY Kolchin SN. Nonunion of the femoral shaft associated with limb shortening treated with a combined technique of external fixation over an intramedullary nail versus the Ilizarov method. Arch Orthop Trauma Surg. (2022) 142:2185–92. doi: 10.1007/s00402-021-03804-4, 33651147

[15] Cho JW Kent WT Kim JK Jeong S-H Sakong S Kim H . Outcome of multi-staged induced membrane technique based on post-debridement cultures for the management of critical-sized bone defect following fracture-related infection. Sci Rep. (2022) 12:22637. doi: 10.1038/s41598-022-26746-2, 36587035 PMC9805441

[16] Nieboer MJ Austin DC Uvodich ME Rogers TH Barlow JD Sanchez-Sotelo J . Acute versus delayed radial head arthroplasty for the treatment of radial head fractures. J Shoulder Elb Surg. (2022) 31:2506–13. doi: 10.1016/j.jse.2022.07.031, 36115618

[17] Yang Y Zou C Fang Y Shakya S. Medium-term clinical results in patients with floating hip injuries. BMC Surg. (2023) 23:40. doi: 10.1186/s12893-023-01927-6, 36803387 PMC9940332

[18] Borzunov DY Kolchin SN Mokhovikov DS Malkova TA. Ilizarov bone transport combined with the Masquelet technique for bone defects of various etiologies (preliminary results). World J Orthop. (2022) 13:278–88. doi: 10.5312/wjo.v13.i3.278, 35317249 PMC8935333

[19] Lin F Yao J Liu Y Qi B. Prepatellar bursa mucosa: an unreported postoperative complication of patellar fracture. Medicine (Baltimore). (2024) 103:e40445. doi: 10.1097/md.0000000000040445, 39533630 PMC11556991

[20] Maeda T Matsumoto T Fujita M Tsubosaka M Kamenaga T Nakano N . Successful total knee arthroplasty for Hoffa and proximal tibial fractures: report of three complex cases after failed osteosynthesis procedures. Am J Case Rep. (2023) 24:e941187. doi: 10.12659/ajcr.941187, 37956116 PMC10658054

[21] Van Wijck SFM Van Diepen MR Prins JTH Verhofstad MHJ Wijffels MME Van Lieshout EMM . Radiographic rib fracture nonunion and association with fracture classification in adults with multiple rib fractures without flail segment: a multicenter prospective cohort study. Injury. (2024) 55:111335. doi: 10.1016/j.injury.2024.111335, 38290909

[22] Lauthe O Gaillard J Cambon-Binder A Masquelet A-C. Induced membrane technique applied to the forearm: technical refinement, indications and results of 13 cases. Orthop Traumatol Surg Res. (2021) 107:103074. doi: 10.1016/j.otsr.2021.103074, 34563733

[23] Guo Z Liu H Luo D Cai T Zhang J Wu J. Application of cortical bone plate allografts combined with less invasive stabilization system (Liss) plates in fixation of comminuted distal femur fractures. Medicina Kaunas. (2023) 59:207. doi: 10.3390/medicina59020207, 36837409 PMC9961610

