# Supplementary material for: An integrated clinical and imaging model for predicting post-traumatic nonunion
Source: Front Med (Lausanne). 2026 Apr 13;13:1784029. doi: 10.3389/fmed.2026.1784029 (PMC13111060; doi:10.3389/fmed.2026.1784029)
Supplement: Supplementary file 1 [file Image_1.pdf]

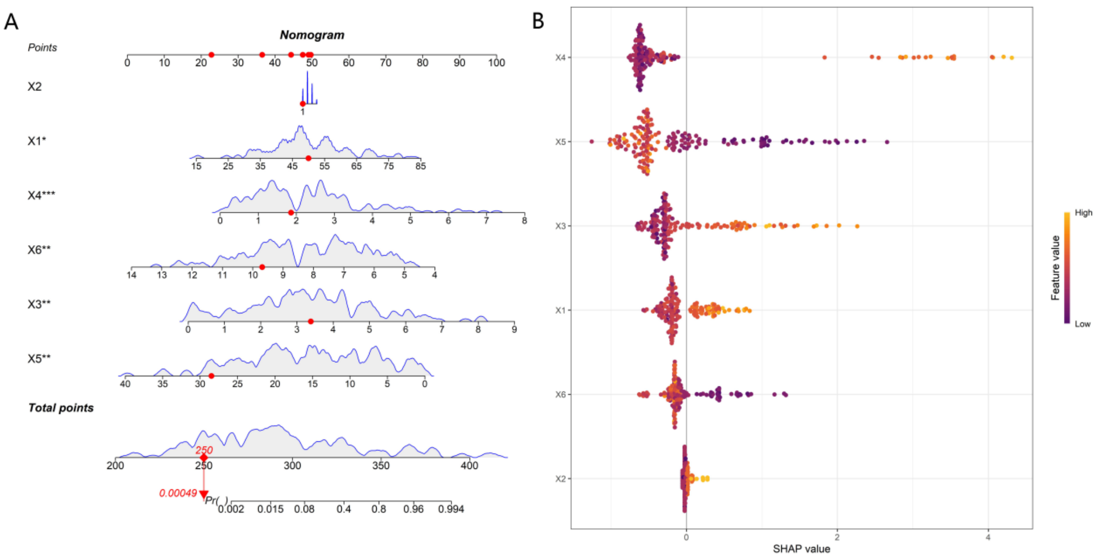

Supplemental Figure 1. Model interpretability analysis (A) Nomogram, (B) SHAP feature importance plot

Note: X1: Injury Severity Index; X2: Maximum Fracture Gap Width ; X3: Cystic Change Volume at Fracture Site ; X4: Callus Volume Growth Rate ; X5: RUST Score
