# Supplementary material for: An integrated clinical and imaging model for predicting post-traumatic nonunion
Source: Front Med (Lausanne). 2026 Apr 13;13:1784029. doi: 10.3389/fmed.2026.1784029 (PMC13111060; doi:10.3389/fmed.2026.1784029)
Supplement: Supplementary file 2 [file Table_1.docx]

**Supplemental Table 1.** Variable assignment table

| Variable | Meaning | Assignment |
| --- | --- | --- |
| X1 | Age | Continuous variable |
| X2 | Injury Severity Index | 1 = Level 1, 2 = Level 2, 3 = Level 3, 4 = Level 4 |
| X3 | Maximum Fracture Gap Width | Continuous variable |
| X4 | Cystic Change Volume at Fracture Site | Continuous variable |
| X5 | Callus Volume Growth Rate | Continuous variable |
| X6 | RUST Score | Continuous variable |
| Y | Post-Traumatic Nonunion | 1=Nonunion Group, 0=Union Group |
